# Resveratrol induces proliferation and differentiation of mouse pre-osteoblast MC3T3-E1 by promoting autophagy

**DOI:** 10.1186/s12906-023-03943-8

**Published:** 2023-04-14

**Authors:** Weiye Cai, Bin Sun, Chao Song, Fei Liu, Zhengliang Wu, Zongchao Liu

**Affiliations:** 1 Department of Orthopaedics and Traumatology, The Affiliated Hospital of Traditional Chinese Medicine of Southwest Medical University, Luzhou, Sichuan China; 2 The People’s Hospital Of Jimo, Jimo, Qingdao China; 3 Tianjin University of Traditional Chinese Medicine, Tianjin, China; 4 Luzhou Longmatan District People’s Hospital, Luzhou, Sichuan Province 646000 China

**Keywords:** Resveratrol, Autophagy, MC3T3-E1, Osteogenic differentiation, Osteoporosis

## Abstract

**Background:**

In mouse, it was discovered that resveratrol (Res) enhanced osteoporosis (OP) by boosting osteogenesis. Besides, Res can also have an impact on MC3T3-E1 cells, which are crucial for the control of osteogenesis and thus increase osteogenesis. Although some articles have discovered that Res enhanced autophagy to promote the value-added differentiation of MC3T3, it is unclear exactly how this affects the process of osteogenesis in mouse. Therefore, we will show that Res encourages MC3T3-E1 proliferation and differentiation in mouse pre-osteoblasts and further investigate the autophagy-related mechanism for this impact.

**Methods:**

(1) MC3T3-E1 cells were separated into blank control group and various concentrations (0.01, 0.1, 1, 10, 100µmol/L) of group in order to determine the ideal Res concentration. In the Res group, Cell Counting Kit-8 (CCK-8) was used to measure the proliferation activity of pre-osteoblasts in mice in each group after resveratrol intervention. Alkaline Phosphatase (ALP) and alizarin red staining were used to gauge the degree of osteogenic differentiation, and RT-qPCR was used to measure the expression levels of Runx2 and OCN in the osteogenic differentiation ability of the cells. (2) In the experiment, four groups were set up: the control group, 3MA group, Res group, and Res + 3MA group. To examine cell mineralization, ALP and alizarin red staining were utilized. RT-qPCR and Western blot detection of cell autophagy activity levels and osteogenic differentiation capacity in each group following intervention.

**Results:**

(1) Resveratrol might increase the number of mice pre-osteoblast, with the impact being most pronounced at 10µmol/L (P < 0.05). The nodules developed substantially more often than in the blank control group, and Runx2 and OCN expressions significantly increased (P < 0.05). (2) In contrast to the Res group, after 3MA purine blocked autophagy, the Res + 3MA group’s alkaline phosphatase staining and the development of mineralized nodules were reduced. Runx2, OCN, LC3II / LC3I expression decreased, p62 expression increased (P < 0.05).

**Conclusion:**

The present study partially or indirectly demonstrated that Res may, through increased autophagy, induce osteogenic differentiation of MC3T3-E1 cells.

**Supplementary Information:**

The online version contains supplementary material available at 10.1186/s12906-023-03943-8.

## Introduction

Osteoporosis (OP) is a common metabolic bone disease in postmenopausal women and the elderly [1]. Osteopenia and increased bone fragility are the main pathological characteristics of OP, and the maintenance of bone mass and bone microstructure depends on the equilibrium point of osteoblast and osteoclast function [2]. There are currently 200 million people living with OP worldwide [3]. Affected by the epidemic, the prevalence of OP in China has also risen significantly, especially women over 40 years old, where it is 4–5 times higher than that of males in the same age group [4]. OP has grown to be a significant global public health issue, placing a significant financial burden on both individuals and society [3]. Therefore, for the creation of novel treatments for OP, it is essential to comprehend the pathophysiology of OP and the cellular and molecular mechanisms involved.

Anti-osteoporosis medications that inhibit bone resorption and encourage bone formation are becoming more common in the development of anti-osteoporosis medications [5]. Traditional hormone treatments are still indispensable in the management of OP, despite the rapid advancement of novel medications [6]. Estrogen has a crucial part in the anti-osteoporosis therapy, which include promoting early osteoblast development, increase collagen and inhibit osteoclast activity [7]. Studies have indicated that estrogen replacement treatment can successfully prevent and cure postmenopausal OP, but it also has some major adverse effects, including an increased risk of breast and uterine cancer [8]. Estrogen replacement therapy is not recommended as a long-term treatment [5]. Therefore, it is crucial to create a medicine that can retain efficacy while reducing adverse effects. Chinese medicine offers a special, comprehensive understanding of treating OP [9]. The phytoestrogens in traditional Chinese medicine are similar to human estrogen in structure and function, and there is no serious adverse reaction. The application of phytoestrogens in OP is increasing [10]. Resveratrol (Res) is an edible phytoestrogen widely found in natural plants such as Polygonum cuspidatum, grapes and peanuts, and has mammalian estrogen-like effects [11]. In vitro and in vivo examinations have demonstrated the way that Res can supplant estrogen in the treatment of OP, and has a protective effect on bone [12–14]. Although Res has been gradually recognized, developed, and clinically used in the treatment of OP [15, 16]. It is still unclear how it exerts its anti-osteoporosis mechanism. As a result, the purpose of this work is to look into the effects of Res on the proliferation and differentiation of pre-osteogenic MC3T3-E1 cells in mice, as well as to investigate its important molecular mechanisms and potential in the therapeutic treatment of postmenopausal OP.

Recent studies have found that autophagy is an important physiological process to maintain the dynamic balance of cells by removing damaged organelles and proteins [17]. Furthermore, assumes a significant part in keeping up with the equilibrium of bone digestion. Autophagy mainly affects the balance of bone metabolism by regulating the differentiation of osteoblasts. The decrease of autophagy level may lead to bone cell dysfunction [18]. Yin X et al. believe that autophagy is a key factor in the pathogenesis of OP [19]. Jiang Y et al. Observed that Res can treat postmenopausal OP by regulating autophagy, but the underlying mechanism has not been fully understood [20]. Mei W et al. have demonstrated that Res is closely associated with pre-osteoblast MC3T3-E1 in the treatment of postmenopausal OP [21]. But the specific mechanism needs to be further explored. Thus, in this experiment, we show that Res encourages the proliferation and differentiation of MC3T3-E1 in rat pre-osteoblasts and further explore the processes behind autophagy.

## Materials and Methods

### Materials

Mouse pre-osteoblast subclone 14 (MC3T3-E1 Subclone 14) was purchased from the Cell Bank of Chinese Academy of Sciences. Res was purchased from Aladdin Reagent Company, China, product number CAS No. 501-36-0. HPLC grade (≥ 94%), α-MEM medium was purchased from HyClone, USA. Fetal bovine serum (FBS) was purchased from Gibco, USA. Runx2 antibody (20700-1-AP), BMP-2 (66383-1-lg), Beclin1 antibody (11306-1-AP), LC3 antibody (14600-1-AP), p62 antibody (18420-1-AP) and β-Actin antibody (66009-1-lg) were purchased from Proteintech. Cell proliferation and toxicity test kit (CCK-8 kit) purchased from Beijing Solarbio Science & Technology Co., Ltd. Alkaline phosphatase (ALP) detection kit (P0321S) and BCIP / NBT alkaline phosphatase chromogenic kit (C3206) were purchased from Shanghai Beyotime Biotechnology Co., Ltd. 3-Methyladenine (3-MA) was purchased from Shanghai source leaf organisms.

## Experimental methods

### Readiness of res

Arrangement 10 mg Res was broken down in 438 µl DMSO to plan 100 mmol/L Res stock arrangement, which was stuffed and put away at -20 °C fridge. The Res stock arrangement was weakened to the accompanying fixations: 0.01, 0.1, 1, 10, 100µmol/L, utilizing α-MEM medium (containing 10% fetal cow-like serum).

### Cell culture

MC3T3-E1 cells were immunized in α-MEM medium (containing 10% fetal ox-like serum) and refined at 37 °C, 5% CO_2_ hatchery. At the point when the cells develop to logarithmic stage, the cells are processed and afterward passaged. The cell development state was firmly noticed. At the point when the cell development combination rate arrived at 80%, the cells were passaged once, and the third and fourth ages of cells were taken for tests.

### CCK-8

The technique was utilized to distinguish the impact of medications on cell expansion function MC3T3-E1 cells developed to 80% conjunction, processed with a stomach related arrangement containing 0.25% trypsin, and made into cell suspension. The cells were cultivated in a 96-well plate with 3000 cells/well and refined in a 5% CO2 hatchery at 37 °C. Following 24 h of cell connection, 200µL of α-MEM culture medium containing various convergences of Res (0.01, 0.1, 1, 10, 100µmol/L) was supplanted, and the cells were refined for 24 and 48 h. 10µL CCK-8 arrangement was added to each well and brooded in the hatchery for 2 h. The OD value at 450 nm was detected by microplate reader.

### ALP staining

MC3T3-E1 cells were blown and mixed evenly, with 12-well plates set to 5 × 10^4^ cells / well evenly spread, divided into blank control group and 0.01, 0.1, 1, 10, 100µmol / L Res group. Cells in each group were treated with different doses of Res for 24 h, and then replaced with osteogenic induction solution containing 50 mg /L ascorbic acid and 10 mol/L β-glycerophosphoric acid for 7 days [22], osteogenic induction solution needs to be changed daily during cell culture. After observing the cell state, the medium was removed, and the cells were washed twice with PBS, immersed in 4% paraformaldehyde for 30 min to fix the cell morphology, washed twice with PBS to remove paraformaldehyde, and stained with NBT-CBIP staining solution at 37 ° C for 30 min in dark. The stained cells were rinsed with distilled water for 3 times, and photographed under a microscope, the staining effect was evaluated by determining the OD value of each group.

### Alizarin red staining

The MC3T3-E1 cells were blown and blended well. The cells were set to 5 × 10^4^/well in 12-well plates and separated into clear benchmark group and 0.01, 0.1, 1, 10, 100µmol/L Res gatherings. Cells in each group were treated with different doses of Res for 24 h, and then replaced with osteogenic induction solution containing 50 mg /L ascorbic acid and 10 mol/L β-glycerophosphoric acid for 21 days [23]. In the wake of noticing the cell express, the medium was eliminated, and the cells were washed two times with PBS, drenched in 4% paraformaldehyde for 30 min to fix the cell morphology, and washed two times with PBS to eliminate paraformaldehyde. Alizarin red staining arrangement with a volume part of 0.2% was ready to stain the cells for 30 min in dim and permitted to remain at room temperature. The stained cells were flushed with refined water for multiple times. General perception of whether there is orange precipitation, tiny perception of whether the cells have mineralized knobs, the OD value of each group was used to assess the staining impact.

### Recognition of protein articulation by western blot

The MC3T3-E1 cells were blown and blended, and the cells were equally spread on a 6-well plate with 1 × 10^5^ cells/well. The cells were partitioned into clear benchmark group and 0.01, 0.1, 1, 10, 100µmol/L Res bunch. After 1 day of culture, cells were cultured in osteogenic induction medium. The MC3T3-E1 cells were washed two times with PBS, and RIPA lysis cushion was added to lyse the cells for protein extraction. The protein was isolated by SDS-PAGE, moved onto PVDF film (The PVDF film was cut into rectangle before antibody hybridization, and covered on the gel according to the position of the target protein.)and brooded with relating essential and auxiliary antibodies. The antibodies utilized incorporate p62, Beclin1, LC3 essential neutralizer and relating auxiliary immune response. The film was set in a chemiluminescence imaging framework, and the outcomes were examined by ImageJ programming to compute the dark worth of each band.

### RT-Qpcr

The outflow of osteogenesis qualities including Runx2, OCN were distinguished through Continuous quantitative PCR (RT-qPCR), and GAPDH was utilized as a source of perspective quality. MC3T3-E1 cells were refined on the frameworks with a thickness of 1 × 10^5^/well in a 6-well plate. After 24 h of hatching with normal α-MEM, the medium was supplanted by osteogenic enlistment medium (50 µg/mL ascorbic acid, 10 mM b- glycerophosphate, and 10^− 8^ M dexamethasone). On days 7 and 14, quality investigation was completed on the cells. More or less, the cells were processed with trypsin, and the complete RNA was extricated utilizing Trizol re-specialist (Invitrogen, US). Right away, cDNA was orchestrated from the extricated mRNA through turn around record response utilizing Prime Content RT reagent Pack (Takara, Japan). Then, Next, RT-PCR was con-ducted utilizing a SYBR Green RT-PCR unit (Takara, Japan) and ABI Stage One Or more Ongoing PCR Framework (Applied Biosystems, US). The examples were rehashed multiple times, and every one of the above tests were per-framed under the maker’s directions. The preliminary groupings of every quality were displayed in Supplementary Table 1.

### Detection of related indicators after adding Autophagy inhibitors

The ideal mediation convergence of Res (10µmol/L) was chosen as the intercession grouping of ensuing tests, and 5 mmol/L 3-Methyladenine (3MA) was added to hinder autophagy. The examination was isolated into control bunch, 3MA bunch (5 mmol/L), Res bunch (10µmol/L), Res bunch (10µmol/L) + 3MA (5 mmol/L).

(1) Basic phosphatase staining was utilized to notice the osteogenic capacity of cells: The cells were equitably plated at 5 × 10^4^ cells/well in a 12-well plate, and 500µL of the comparing arrangement was added to each well as per the 2.4.

(2) Perception of mineralized knobs by alizarin red staining: The cells were uniformly plated at 5 × 10^4^ cells/well in a 12-well plate, and 500µL of the relating arrangement was added to each well as per the 2.5.

(3) Identification of Beclin1, LC3 and p62 protein articulation in cells by Western smudge: The cells were equally plated in 6-well plates at 1 × 10^5^ cells/all things considered, and 100µL of the relating arrangement was added to each well as per the 2.6.

### Data analysis

Measurable investigation SPSS 20.0 programming was utilized for factual examination. The trial information were communicated as X ± S. All examinations were rehashed multiple times. The mean correlation between the two gatherings was performed by free examplet test. The mean examination between numerous gatherings was performed by one-way investigation of change. Further pairwise examination was performed by LSD test. P < 0.05 was viewed as measurably huge.

## Results

### Res promotes the proliferation of MC3T3-E1 cells

**Fig. 1:**
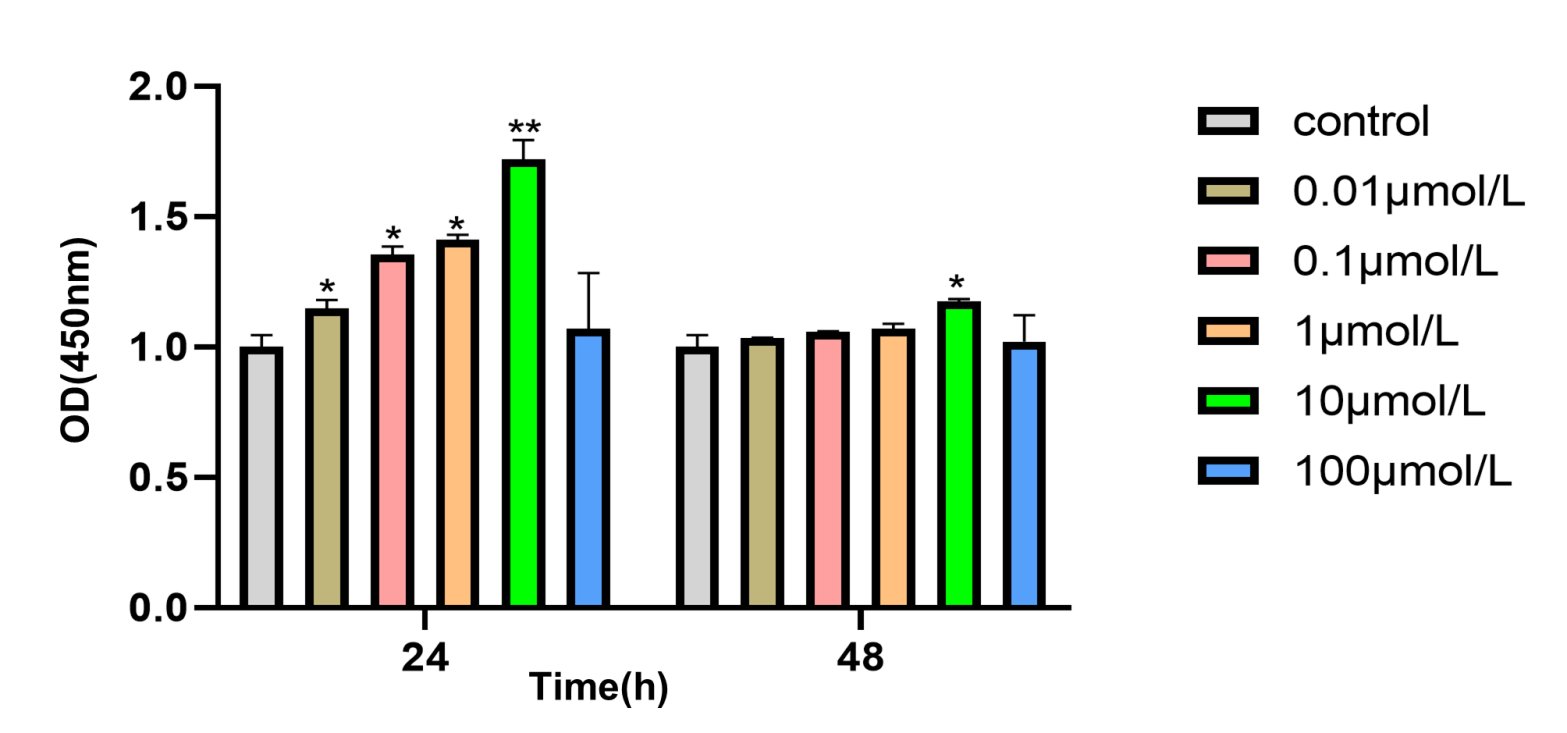
Effect of Res on viability of MC3T3-E1 cells MC3T3-E1 cells were treated with 0.01,0.1,1,10 and 100µmol/L Res for 24 and 48 h, and cell proliferation was detected by CCK-8. *P＜0.05 vs. control

Firstly, the effect of Res on the proliferation of mouse MC3T3-E1 was investigated. The MC3T3-E1 cells were treated with 0.01, 0.1, 1, 10, 100 µ / L Res for 24 and 48 h Respectively, as shown in Fig. 1. Compared with the control group, the proliferation of MC3T3-EI cells was increased after the cells were treated with 0.01, 0.1, 1, 10µmol / L Res for 24 and 48 h. Figure 1 shows that the cellular value-added capacity at 48 h is lower than that at 24 h, besides, due to the mild toxicity of resveratrol, prolonged cell growth at high concentrations may result in a small amount of cytotoxicity and cell death, therefore we assume that resveratrol’s intervention on cells is most effective during 24 h. Furthermore, the effect was the most obvious at 10µmol / L, but the proliferation was inhibited when the concentration reached 100µmol / L (P ＜ 0.05), indicating that Res could promote the proliferation of osteoblasts at an appropriate concentration (CCK8 results are shown in Fig. 1).

### Res promotes osteogenic differentiation of MC3T3-E1 cells

**Fig. 2:**
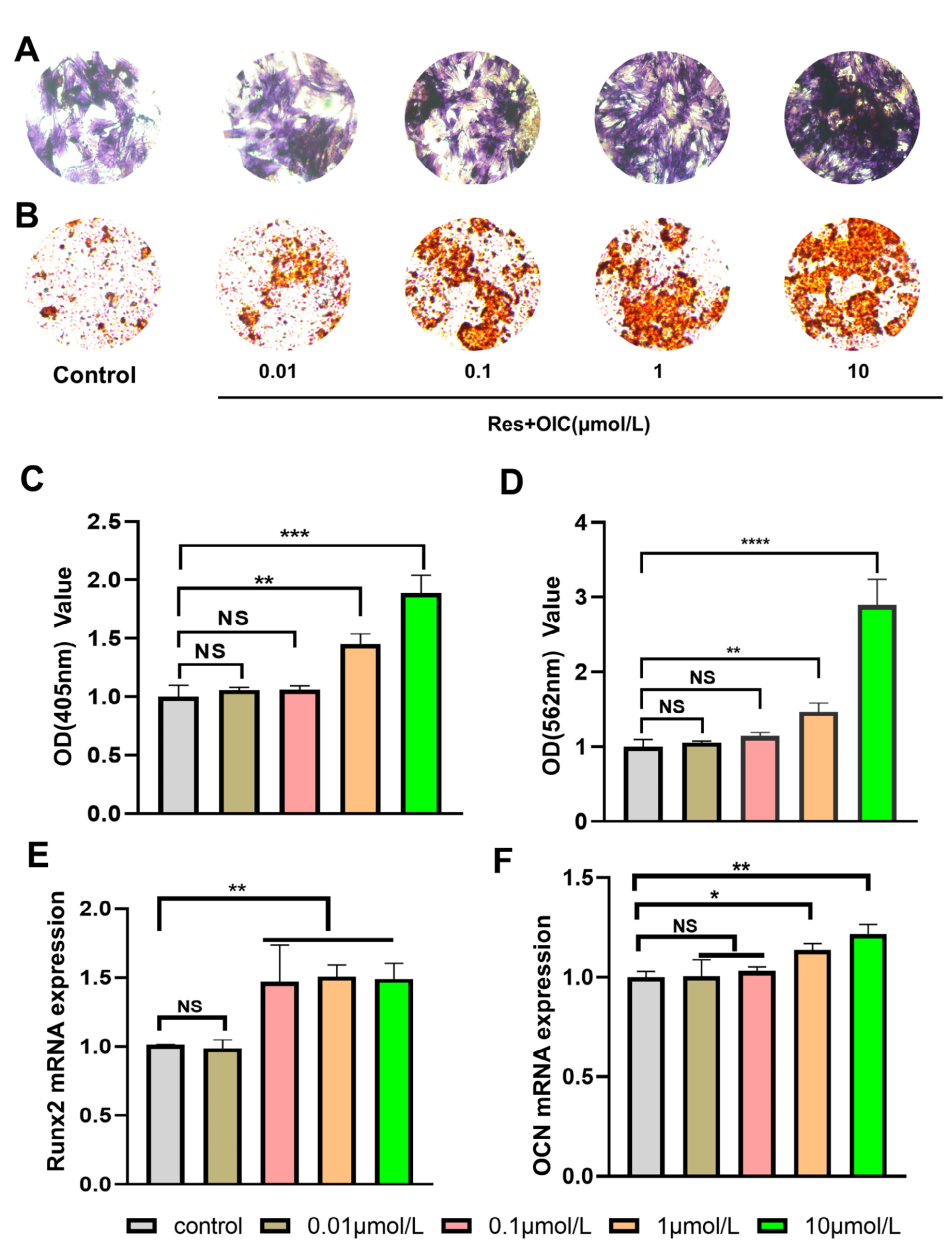
Effect of Res on osteogenic differentiation of MC3T3-E1 cells **(A)** ALP staining in each group (x100). Compared with the control group, staining in the 10µmol/L Res treatment group was significantly increased. **(B)** The alizarin red staining of each group(x100). Compared with the control group the formation of mineralized nodules was significantly increased in the 10µmol/L Res treatment group. **(C)** The relative expression of ALP levels. *P < 0.05, ***P < 0.01 vs. control. **(D)** The relative expression of calcium nodules levels. *P < 0.05, ***P < 0.01 vs. control. **(E)** The expression level of Runx2 mRNA by RT-qPCR; **(F)** The expression level of OCN mRNA by RT-qPCR. *P < 0.05, ***P < 0.01 vs. control

Alkaline phosphatase (ALP) is an early marker of osteogenic differentiation. The higher the ALP activity, the more mature the differentiation [24]. After 7 days of culture in osteogenic induction medium, the result of alkaline phosphatase staining is shown in the Fig. 2A. With the increase of Res concentration, the degree of ALP staining increased gradually. Contrasted and the control group, the staining impact of 10µmol/L Res treatment bunch was the clearest, it is suggested that osteoblast differentiation is the most mature under the intervention of 10µmol/L Res. The results of ALP activity assay are shown in the Fig. 2C. Compared with the control group, the expression levels of ALP were significantly increased after 0.01, 0.1, 1 and 10µmol / L Res treatment, and the expression level of ALP was the highest in 10µmol / L Res group (P = 0.0006; t = 8.601, df = 4).

Extracellular matrix mineralization is considered to be one of the important markers of late maturation of osteoblast differentiation [25]. Following 21 days of osteogenic enlistment culture, the results of alizarin red staining are shown in Fig. 2B: With the increment of intriguing earth focus, the formation of mineralized nodules increases gradually. And the mineralization staining of 10µmol / L Res treatment group was the most obvious. Figure 2D shows the results of alizarin red S staining. Compared with the control group, the formation of mineralized nodules was increased in 1 and 10µmol/ L Res treatment groups (P = 0.0058; t = 5.377, df = 4), which indicated that Res could promote the formation of calcium nodules in MC3T3-EI osteoblasts and thus promote osteogenic differentiation.

Runx2 also plays an important role in the early stage of osteogenic differentiation of cells. It is a downstream transcription factor of many osteogenic related pathways. Reduction will lead to a decrease in the level of osteogenic differentiation [26]. OCN is a component of the extracellular matrix of bone, is one of the main signs of osteoblast differentiation and maturation into the mineralization period, is considered to be a late marker of osteoblast differentiation and maturation [27, 28]. The q-PCR results are shown in the Fig. 2E-F: Contrasted and the benchmark group, there was no significant difference in the 0.01µmol / L Res treatment group (P = 0.5059;t = 0.7299, df = 4). The expression levels of Runx2 gene in 0.1, 1, 10µmol / L Res groups were significantly enhanced (P = 0.018；t = 7.423, df = 4), and the expression level of 10µmol / L treatment group was the most significant. The 10µmol / L Res group could significantly increase the expression of OCN gene (P = 0.025；t = 6.793, df = 4), and the difference was statistically significant. The outcomes further demonstrated that Res could advance the osteogenic differentiation of MC3T3-E1 cells.

### Res improved autophagy of MC3T3-E1 cells

**Fig. 3:**
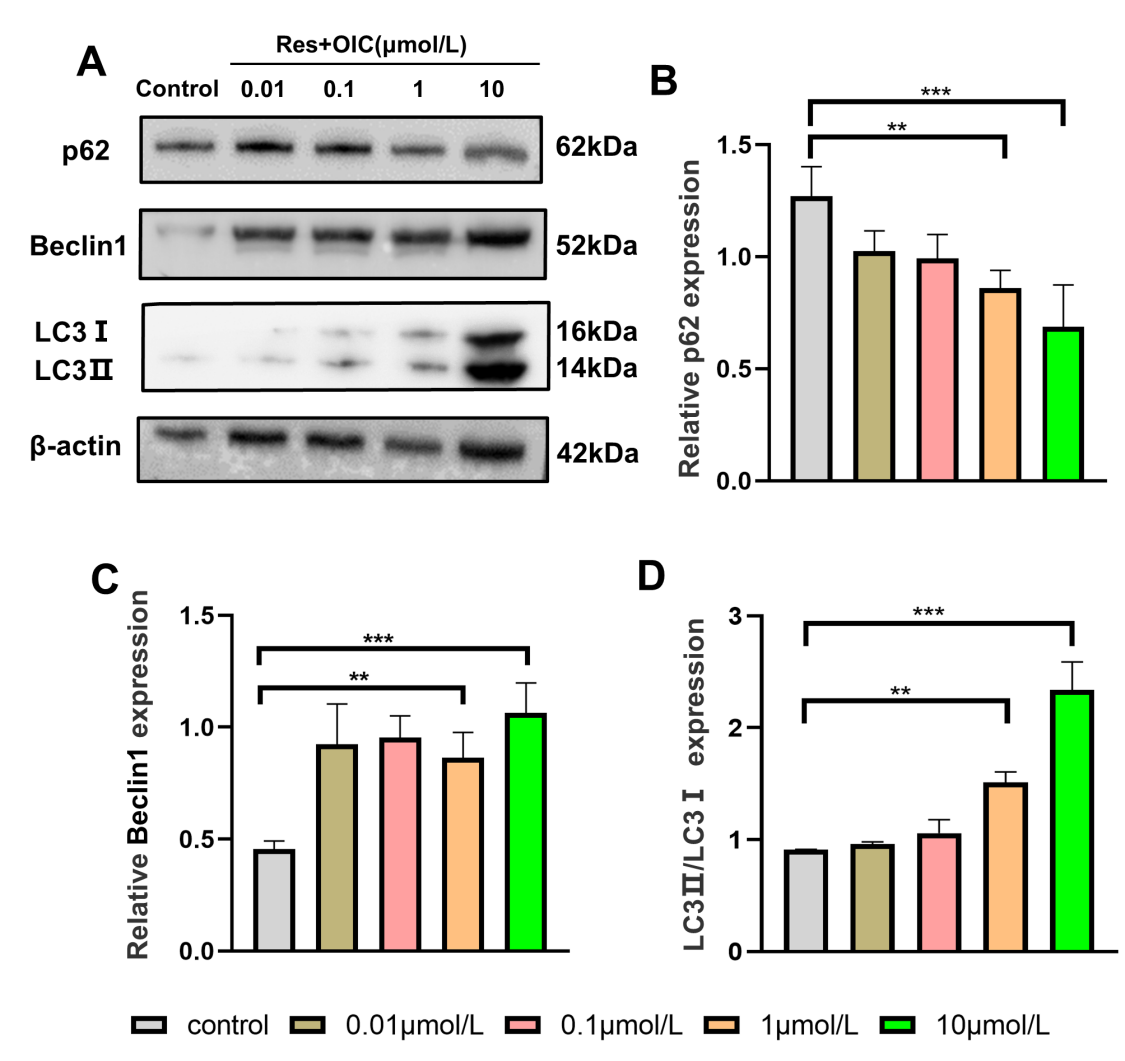
Effect of Res on the expression of autophagy related proteins of MC3T3-E1 cells **(A)** The electrophoresis of protein expression. **(B-D)** The relative protein expression levels. *P < 0.05, ***P < 0.01 vs. control

As shown in the Fig. 3A: Compared with the control group the relative expression of autophagy protein Beclin1 and LC3 protein in the 0.1, 1, 10µmol / L Res group increased in a concentration-dependent manner. The expression of p62 protein decreased gradually (P = 0.0038; t = 6.035, df = 4；P = 0.0016；t = 7.549, df = 4). The increase of LC3II / LC3I ratio and the decrease of p62 expression suggested that Res could enhance the autophagy of MC3T3-E1 cells, and the effect was most obvious when the concentration was 10µmol / L (P = 0.0006；t = 9.924, df = 4). According to the above experimental results 10µmol / L Res was the optimal concentration, so we chose 10µmol / L as the intervention concentration for subsequent experiments (Fig. 3B-D).

### Res attenuates osteogenic differentiation in MC3T3-E1 cells after autophagy inhibition

**Fig. 4:**
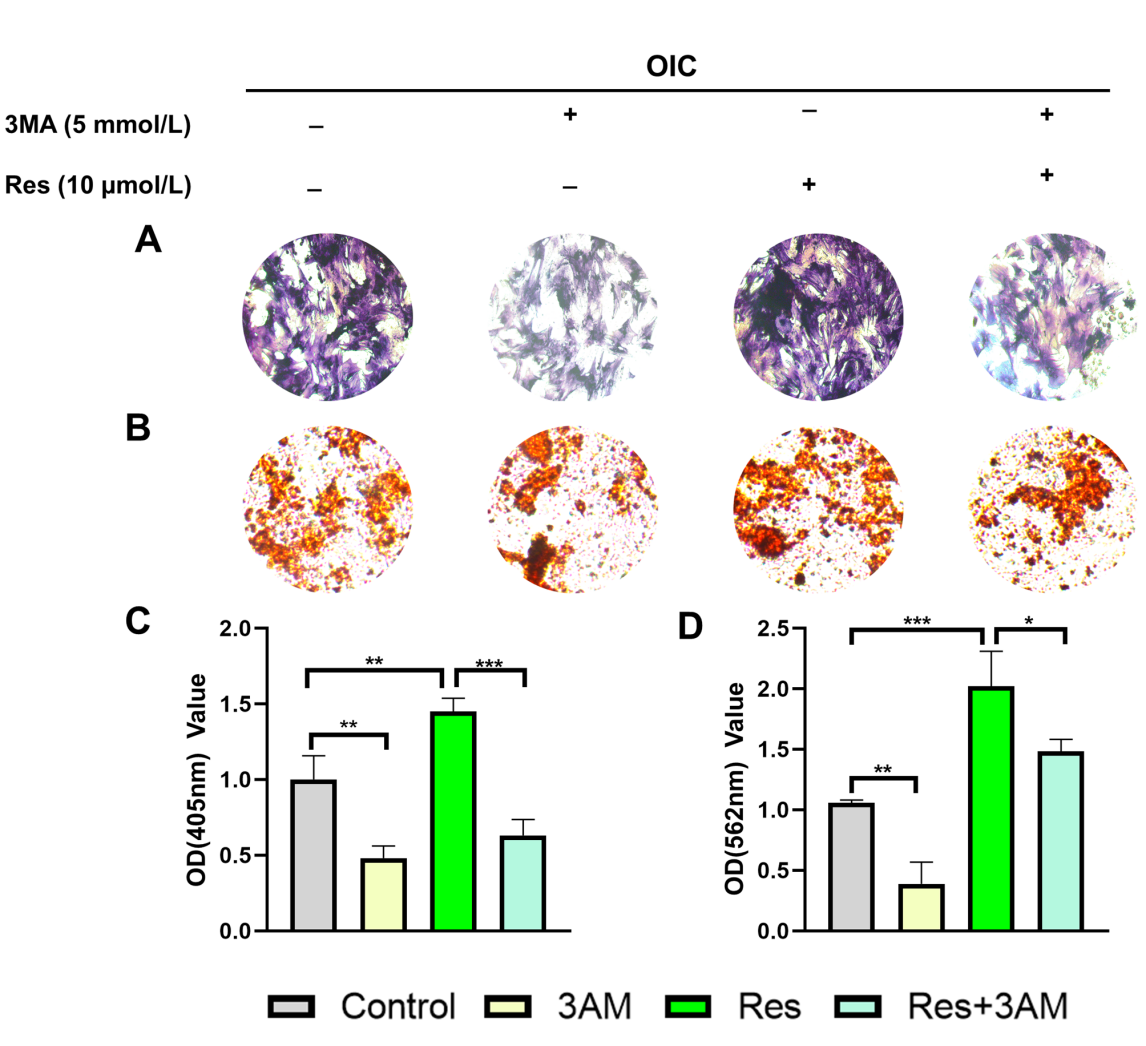
Effect of inhibition of autophagy on the expression of osteogenic differentiation-related level **(A)** ALP staining in each group (x100). Compared with the Res group, staining was significantly reduced the Res + 3MA group. **(B)** The alizarin-red staining for each group(x100). Compared with the Res group, the formation of mineralized nodules was significantly reduced in the Res + 3MA group. **(C)** The relative expression of ALP levels. *P < 0.05, ***P < 0.01 vs. Res group. **(D)** The relative expression of calcium nodules levels, *P < 0.05, ***P < 0.01 vs. Res group

In order to further study whether Res enhances the osteogenesis of MC3T3-E1 cells through autophagy, the resulting tests were isolated into four gatherings: Control group, 3-methyladenine (3MA) group, Res group, Res + 3MA group. The above four groups were cultured in osteogenic differentiation medium at the same time. After 7 and 21 days, the results of ALP and alizarin red staining were as follows: compared with the control group, the degree of ALP and mineralization staining was significantly reduced after adding 3MA, indicating that 3MA inhibited the expression of ALP and the mineralization level of extracellular matrix, while the addition of Res could significantly promote the staining level of ALP and alizarin red, and the combined use of 3MA significantly inhibited the promoting effect of Res. The effect of Res on osteogenic differentiation of MC3T3-E1 cells was significantly decreased (Fig. 4A-B). Figure 4 C-D results showed that after 3MA inhibited autophagy, the expression of ALP and the formation of mineralized nodules in Res + 3MA group were significantly lower than those in Res group (P = 0.0005;t = 10.42, df = 4；P = 0.0377；t = 3.059, df = 4), which was consistent with the results of alizarin red staining. The above results indicate that Res can enhance the level of osteogenic differentiation and mineralization, and this effect of promoting osteogenic differentiation can be blocked by autophagy blockers. In summary, the ability of Res to induce osteogenic differentiation is achieved through autophagy regulation.

### Res attenuated autophagy expression in MC3T3-E1 cells after autophagy inhibition

**Fig. 5:**
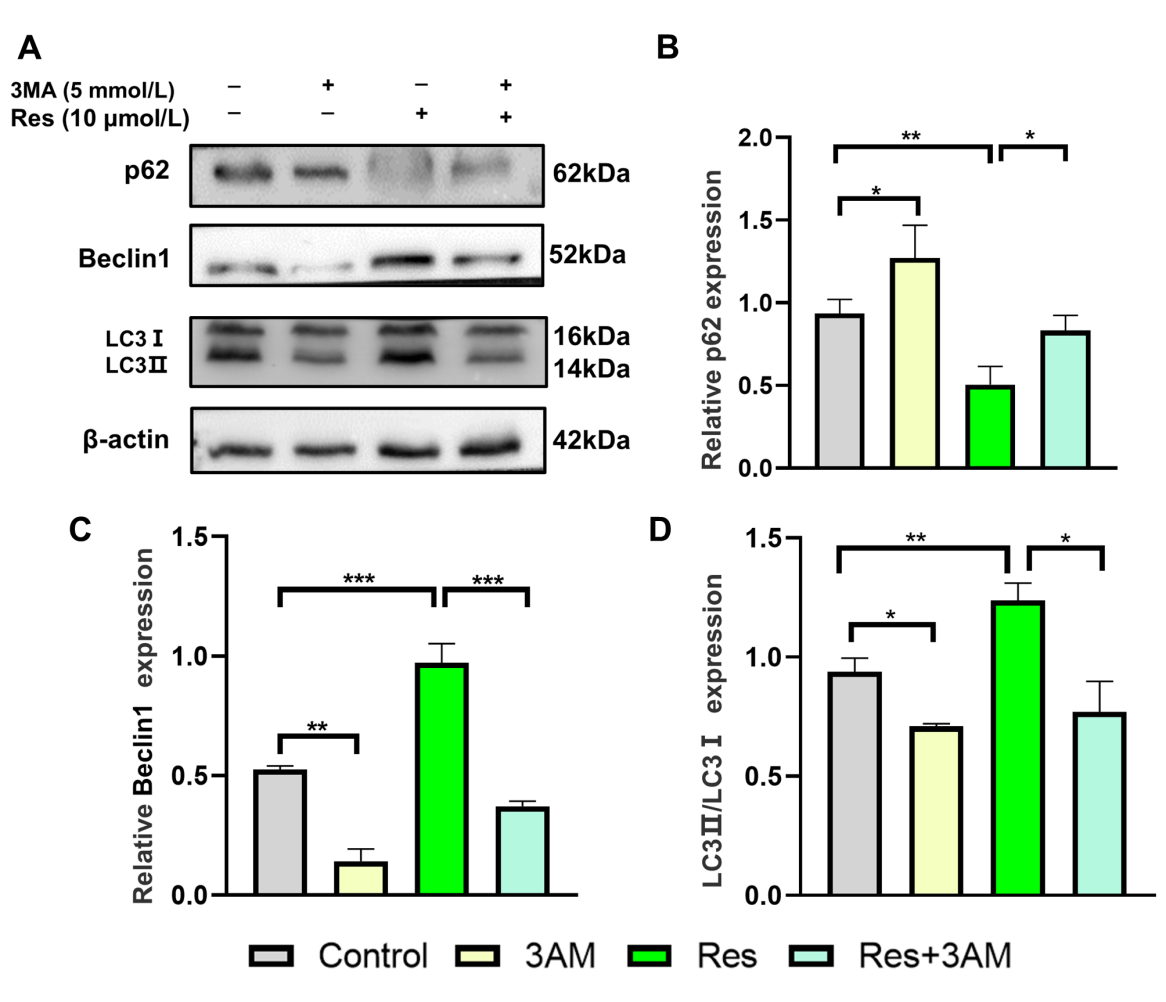
Effect of inhibition of autophagy on the expression of autophagy related proteins **(A)** The electrophoresis of protein expression. **(B-D)** The relative protein expression levels. *P < 0.05, ***P < 0.01 vs. Res group

During autophagy, the microtubule associated protein light LC3I is converted into LC3II to initiate autophagy body formation and autophagy, whereas the substrate for autophagic degradation of p62 binds to LC3 on the membrane of autophagosome during autophagy formation and is subsequently degraded by autophagosome [29, 30]. Therefore, the expression of LC3II / I was positively correlated with the level of autophagy, while the expression of p62 was inversely proportional to the activity of autophagy. In order to investigate the effect of Res on the autophagy level of MC3T3-E1, we used the classical autophagy inhibitor 3MA alone or in combination to inhibit the autophagy level. Western blot experiments in Fig. 5 showed that compared with the control group, 3MA significantly increased the expression of p62 and decreased the expression of Beclin1 and LC3 (P = 0.0166;t = 3.963, df = 4；P = 0.0052；t = 6.308, df = 4；P = 0.0470；t = 2.3645, df = 4), Res significantly increased the expression of Beclin1 and LC3 and decreased the expression of p62 (P = 0.0007；t = 9.451, df = 4；P = 0.0096；t = 4.657, df = 4；P = 0.0032；t = 3.963, df = 4), indicating that Res can effectively promote autophagy in MC3T3-E1 cells. Compared with Res group, LC3II / I ratio and Beclin1 expression decreased in Res + 3MA group. The expression of p62 protein increased, which indicated that 3MA could inhibit the promoting effect of Res on autophagy in MC3T3-E1 cells (P = 0.0166；t = 3.963, df = 4；P = 0.0002；t = 12.50, df = 4; P = 0.042；t = 4.587, df = 4).

## Discussion

OP is a metabolic bone disease in which osteoclasts are more active in bone resorption than osteoblasts in bone formation [31]. Imbalance between osteogenesis and osteoclasts is more pronounced in postmenopausal women due to estrogen deficiency [32]. Res promotes osteoblast differentiation in ovariectomized rats by regulating autophagy, study finds [33]. In addition, another study found that Res promoted the proliferation and induced autophagy of osteoblast precursor cells MC3T3-E1 [34]. However, the underlying mechanism of Res in the treatment of postmenopausal OP is not fully understood. Therefore, this study aims to further demonstrate that Res may promote the proliferation and differentiation of mouse pre-osteoblast MC3T3-E1 by enhancing autophagy.

To clarify how Res works to treat OP, we decided the impact of Res on the expansion and separation of pre-osteoblast MC3T3-E1 cells and the job of autophagy. Firstly, we demonstrated that Res could promote the proliferation of MC3T3-EI cells by CCK8 assay, this is consistent with the results of Liu Xu and others [34]. Secondly, we believe that Res can promote osteoblast differentiation, and ALP and alizarin red staining results confirmed our hypothesis: Res treatment increases the formation of mineralized nodules, and we further verified that it promoted the formation of calcium nodules in MC3T3-EI osteoblasts and promoted osteogenic differentiation. Ma et al. found that Res reversed the inhibition of osteoblast differentiation induced by lipopolysaccharide, the use of Res-loaded polylactic acid also had an inhibitory effect on osteoclast differentiation, confirming our concept [35].

Autophagy is eukaryotic cells through autophagy to intracellular abnormal proteins, damaged or aging organelles, pathogens and other degradation, its metabolites such as amino acids, nucleotides and re-used by cells, material synthesis, energy metabolism and other life activities [36]. Autophagy mainly plays an adaptive role in protecting organisms from various pathological effects, including infection, cancer, aging, etc. [37]. Thus, autophagy is an important mechanism to maintain the stability of intracellular environment. In recent years, the role of autophagy in bone formation has been gradually discovered [38]. Nollet M believes autophagy is activated during osteoblast differentiation [39]. Nuschke ‘s study showed that autophagy increased significantly in human mesenchymal stem cells at the early stage of osteogenic differentiation, and decreased significantly after differentiation into mature osteocytes, suggesting that autophagy plays an important role in osteogenesis [40]. Further studies have shown that autophagy can promote osteogenic differentiation and improve mineralization by regulating NF-κB [41] and TNFSF11 / RANKL[39] signaling pathways, which is conducive to bone formation. In addition, autophagy reduces oxidative stress during aging, making autophagy an important factor in OP [42]. Therefore, we speculate that Res enhances the proliferation and osteogenic differentiation regulation of MC3T3-E1 cells may be related to autophagy. The changes of autophagy-related proteins p62, LC3 and Beclin1 in pre-osteoblast MC3T3-E1 and autophagy flux were detected by experiments. And the results showed that the relative expression of autophagy associated proteins Beclin1 and LC3 increased in a concentration dependent manner in 10µmol / L Res group, while the expression of p62 protein decreased gradually. LC3II/LC3I ratio increased and p62 expression decreased. It is suggested that Res can enhance the autophagy ability of MC3T3-E1 cells. LC3II is a classic marker of autophagy maturation, and its expression is positively correlated with autophagy level [43]. Latest research also suggests that increased cytoplasmic LC3II is caused by inhibition of autophagy [44]. Therefore, the experimental results of increased LC3II / LC3I ratio can confirm that Res enhances the proliferation and osteogenic differentiation of MC3T3-E1 cells by regulating autophagy. To further verify the autophagy process, we used autophagy inhibitor 3MA to inhibit autophagy. The outcomes showed that when autophagy was repressed, Res advanced osteogenic separation of MC3T3-E1 cells was turned around.

In summary, we found that Res can promote osteoblast differentiation and promote bone formation through cell experiments. The potential mechanism is to enhance the differentiation of MC3T3-E1 cells into osteoblasts by promoting autophagy. Our work will add to the mechanistic study and clinical treatment of postmenopausal OP therapy. Of course, there are still some limitations in the experiment. The experiment only did cell verification, and lacked certain in vivo experimental verification. We will further study the relevant mechanisms later.

## Conclusion

The present study partially or indirectly demonstrated that Res may, through increased autophagy, induce osteogenic differentiation of MC3T3-E1 cells.

## Electronic supplementary material

Below is the link to the electronic supplementary material.

**Additional file 1: Supplementary Table 1.** Primer sequences of osteogenesis-related genes.

**Additional file 2.**

## Data Availability

The datasets used and/or analysed during the current study are available from the corresponding author on reasonable request.

## Abbreviations

Res: Resveratrol
OP: Osteoporosis
CCK-8: Cell Counting Kit-8
ALP: Alkaline phosphatase
FBS: Fetal bovine serum
3-MA: 3-Methyladenine
