# Supplementary material for: Resveratrol induces proliferation and differentiation of mouse pre-osteoblast MC3T3-E1 by promoting autophagy
Source: BMC Complement Med Ther. 2023 Apr 14;23:121. doi: 10.1186/s12906-023-03943-8 (PMC10103476; doi:10.1186/s12906-023-03943-8)
Supplement: Supplementary file 1 — Additional file 1: Supplementary Table 1. Primer sequences of osteogenesis-related genes. [file 12906_2023_3943_MOESM1_ESM.docx]

Supplementary Table 1. Primer sequences of osteogenesis-related genes.

| Gene | Forward primer (5′-3′) | Reverse primer (5′-3′) |
| --- | --- | --- |
| RUNX2 | GCGCATTCCTCATCCCCAGTA | AGTTCTGAAGCACCTGCCTG |
| OCN | ACATGCAGTTCCGAAAGGTCA | GCAGGTTCTTTAGGACTTCTCGTT |
| GAPDH | ATCAAGAAGGTGGTGAAGCA | AGACAACCTGGTCCTCAGTGT |
