# Supplementary figures and images for: Resveratrol induces proliferation and differentiation of mouse pre-osteoblast MC3T3-E1 by promoting autophagy
Source: BMC Complement Med Ther. 2023 Apr 14;23:121. doi: 10.1186/s12906-023-03943-8 (PMC10103476; doi:10.1186/s12906-023-03943-8)

Fig 3A

P62

1-1

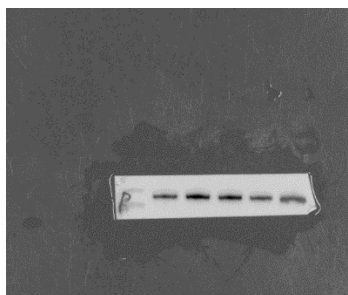

2-2

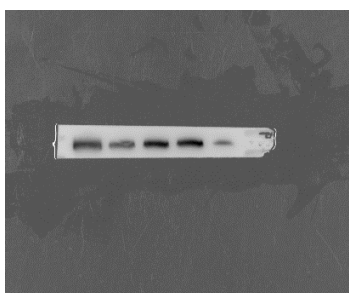

Beclin1

1-1

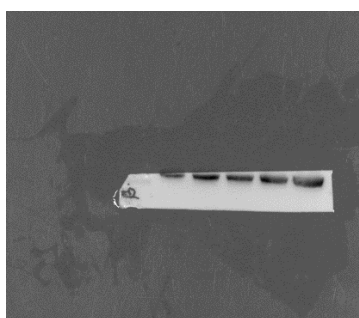

2-2

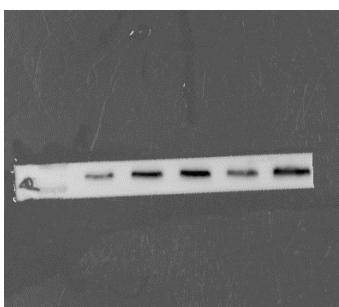

LC3

1-1

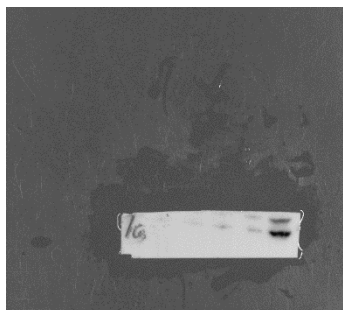

2-2

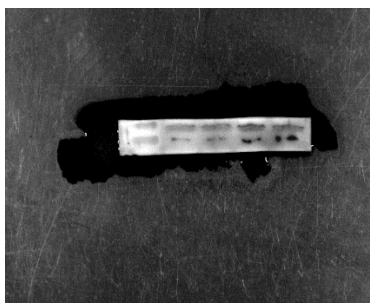

$\beta$ -actin

1-1

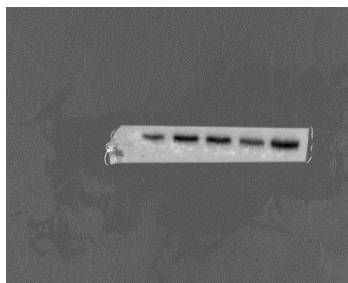

2-2

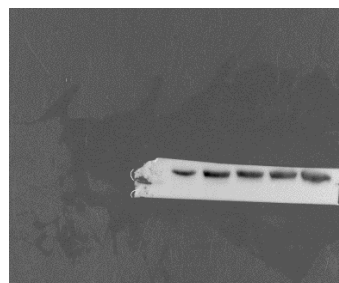

3-3

P62

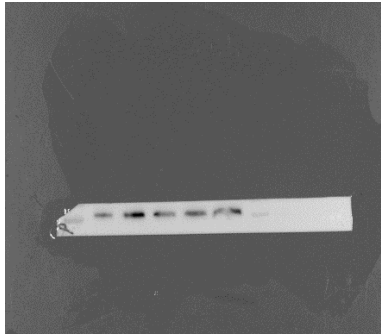

Beclin1

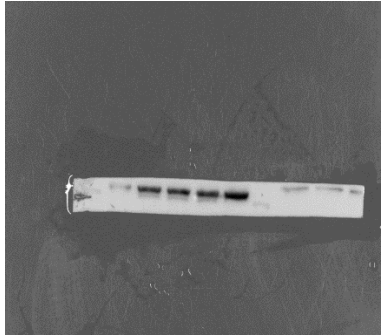

LC3

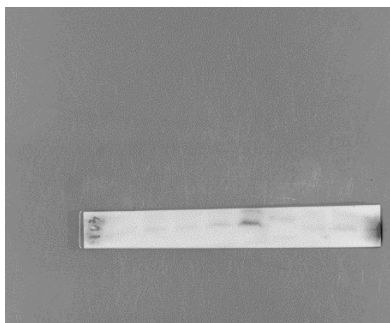

$\beta$ -actin

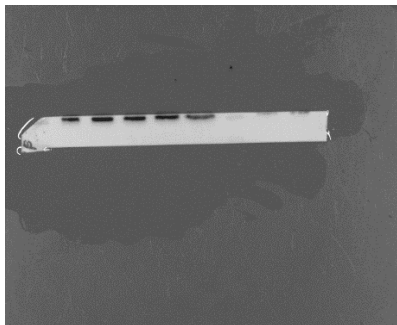

Fig5.

P62

1-1

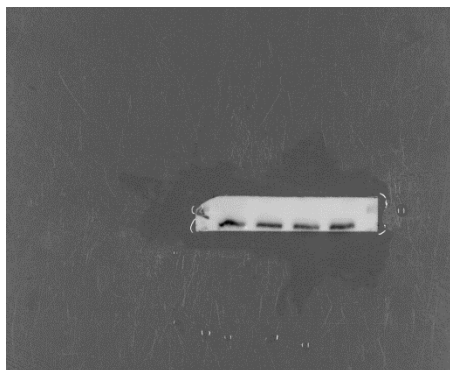

2-2/3-3

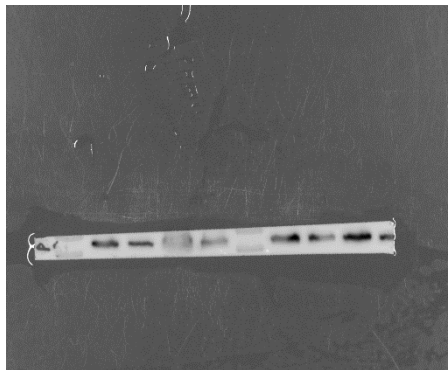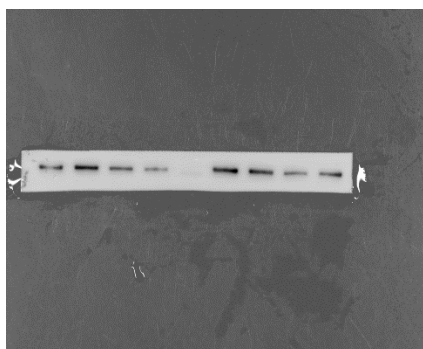

Beclin1

1-1

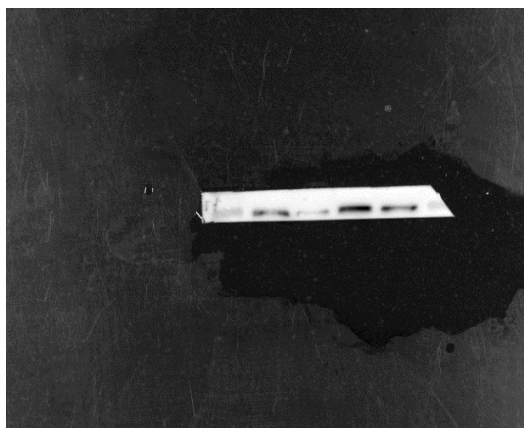

1-2/1-3

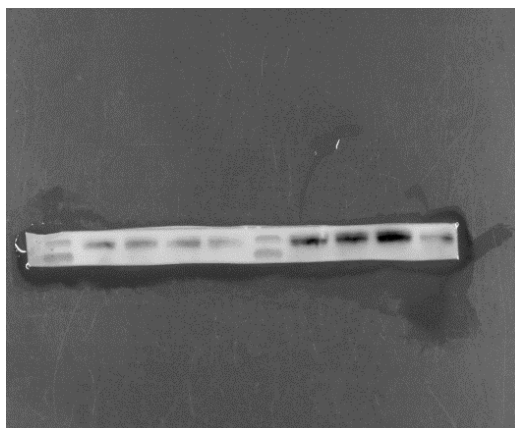

LC3

1-1

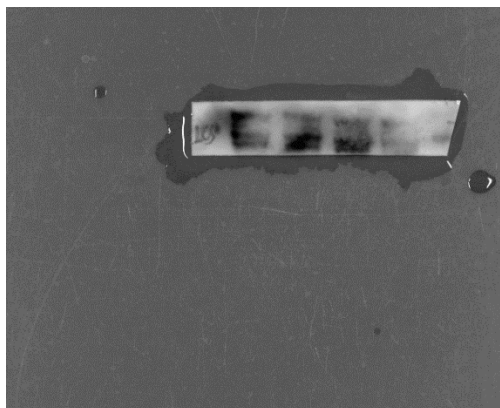

1-2/1-3

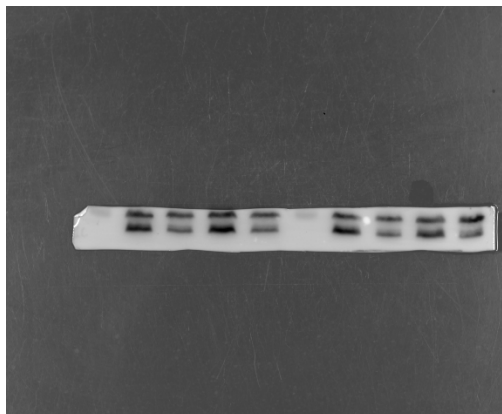

$\beta$ -actin

1-1

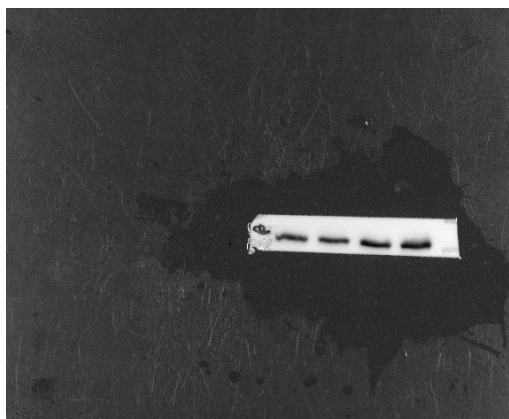

1-2/1-3

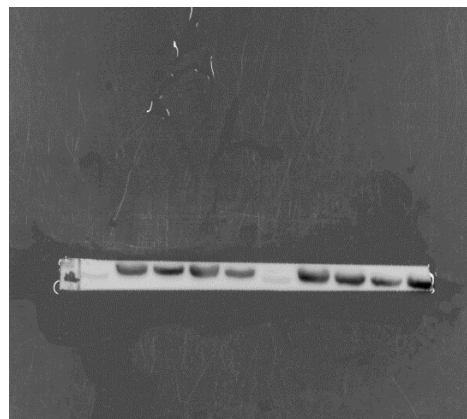

Supplement: Supplementary file 2 — Additional file 2. [file 12906_2023_3943_MOESM2_ESM.pdf]
